# Celiac Disease Masquerading as Arthralgia

**DOI:** 10.7759/cureus.26387

**Published:** 2022-06-28

**Authors:** Shista Priyadarshini, Ayesha Asghar, Sohaib Shabih, Vineela Kasireddy

**Affiliations:** 1 Internal Medicine, Guthrie Robert Packer Hospital, Sayre, USA; 2 Internal Medicine, Aga Khan University Hospital, Karachi, PAK; 3 Internal Medicine, Patel Hospital, Karachi, PAK; 4 Department of Hematology and Oncology, Capital Region Medical Center, Jefferson City, USA

**Keywords:** iron deficiency anaemia, atypical presentation of celiac disease, anemia, arthralgia, non classical celiac disease

## Abstract

Celiac disease is an immune-mediated disorder triggered by dietary gluten. It classically presents with gastrointestinal symptoms. It may also present with atypical manifestations like anemia, arthritis, infertility, or other neurological symptoms. However, arthralgia as a sole manifestation of celiac disease is a rare clinical scenario. Even though the clinical spectrum of celiac disease is broad, prompt diagnosis and management exert a protective effect against complications of celiac disease. We want to highlight and expand on the existing knowledge on atypical presentations about celiac disease.

## Introduction

Celiac disease is an immunologically mediated disorder that is triggered by gluten and gliadin in the diet. The global prevalence is about 1% [1]. The incidence is on the rise, as per recent studies [2]. It usually presents as diarrhea as well as malabsorptive features. The atypical manifestations are many, ranging from anemia, arthritis, infertility to neurological symptoms [3,4]. This manuscript aims to create awareness about arthralgia as an atypical presentation of celiac disease.

## Case presentation

A 52-year-old Caucasian man presented to the clinic with complaints of swelling and pain in his bilateral knee and ankle joints. There was no significant past medical history. He denied any shortness of breath, chest pain, fatigue, skin rash, tick bites, or leg claudication. He was hemodynamically stable. On examination, the knees and ankles were swollen but non-tender. Laboratory investigations showed severe anemia with a hemoglobin of 7.2 g/dl. He was advised to take supportive measures such as limiting his salt intake and keeping his legs elevated at rest. There was no improvement in joint pain or swelling. Further workup showed serum iron at 22 mcg/dl, ferritin 4.3 ng/ml, and a total iron-binding capacity of 460 mcg/dl, consistent with iron deficiency (Table 1).

**Table 1:** Laboratory investigations Units - gm/dl: gram/deciliter; mcg/dl: microgram/deciliter; ng/ml: nanogram/milliliter

Laboratory tests	Values	Normal range
Hemoglobin (gm/dl)	7.2	13–15
Serum iron (mcg/dl)	22	59–158
Ferritin (ng/ ml)	4.3	30–400
Total iron binding capacity (mcg/dl)	460	149–505

Though asymptomatic, he was started on intravenous iron for severe anemia. This was discontinued due to an allergic reaction. Given his age, it prompted endoscopy and colonoscopy to look for iron deficiency anemia and gastrointestinal malignancy. The colonoscopy was unremarkable. Endoscopy showed flattened mucosa in the duodenum. Duodenal biopsy showed marked villous blunting to flattening with increased intraepithelial lymphocytes, compatible with celiac disease. Transglutaminase and anti-endomysial antibodies were also positive. Interestingly, the patient never had any abdominal symptoms associated with gluten intake. He was advised to start a gluten-free diet and, subsequently, his joint pain symptoms resolved. He was started on iron supplements and continued on a gluten-free diet as well. Subsequently, he was followed at a hematology clinic for iron deficiency anemia and labs showed normalization of hemoglobin levels to 11.0 g/dl within a month. A three-month follow-up showed hemoglobin levels improved to 15.3 g/dl.

## Discussion

Celiac disease is an immunologically mediated enteropathy caused by impaired tolerance to gluten. It affects 1-2% of the population in the west and the incidence is continuously increasing [2]. Typically, it presents with symptoms of malabsorption such as weight loss, diarrhea, steatorrhea, or abdominal distension. However, there are reports of increasing trends in extraintestinal manifestations of celiac disease. The existing literature mentions dermatologic, endocrine, skeletal, hepatic, hematological, thrombophilia, fertility, dental, psychiatric, and behavioral abnormalities [3]. Extraintestinal manifestations may include anemia, osteopenia, papulovesicular rash, paresthesia, ataxia, muscle weakness, numbness, tingling, infertility in women and impotence in men [4].

The most commonly reported rheumatological symptoms in celiac disease include polyarthralgia, early morning joint stiffness, back pain, and sacroiliitis [5]. But arthralgias as a sole presentation of celiac disease is a rare occurrence [6,7]. Significant improvement has been reported in individuals with rheumatological manifestations who consume a gluten-free diet. The exact underlying mechanism is unclear, but dietary gluten has pro-inflammatory and pro-oxidative effects. It has also been shown to dampen regulatory T-cell activity [8]. These characteristics might explain some of the anti-inflammatory effects of gluten withdrawal on the joint manifestations in celiac disease and the improvement of joint pain in our patients.

As per guidelines, serum Immunoglobulin-A anti-tissue transglutaminase antibody (IgA TTG) is the preferred initial test for workup of celiac disease. In the case of IgA deficiency, measuring the IgG class of TTG is recommended [9]. The main diagnostic criteria continue to be duodenal biopsy findings, which include intraepithelial lymphocytes, the presence of crypt hyperplasia, and inflammatory infiltrates in the lamina propria. These findings are mainly seen when the patient is following a regular diet. If a histological examination yields equivocal results, it is useful to proceed with HLA typing as HLA DQ-2 and HLA DQ-8 markers are seen in 90-95% of cases of celiac disease [10].

The mainstay of treatment of celiac disease is the avoidance of gluten in the diet. Some of the foods rich in gluten include wheat, barley, rye, green spelt, and oats. However, complete abstinence from gluten-containing grain products is somewhat challenging because wheat flour is an integral part of the American diet. Patients must be followed clinically with serologic testing (mainly tissue transglutaminase and deamidated gliadin peptide) to determine response to treatment. Corticosteroids may be used in celiac disease patients who do not respond to gluten-free diets [11]. When there is severe iron deficiency anemia, parenteral iron supplementation is recommended for two to three months to hasten the recovery time.

## Conclusions

Classification of celiac disease as a gastrointestinal disease with gastrointestinal manifestations leads to underdiagnosis or misdiagnosis of asymptomatic and atypically presenting patients. It is important to include serological screening tests systematically in the evaluation of adult patients who present with unexplained iron deficiency anemia and non-specific symptoms.
